# Cholesterol Paradox in Older People with Type 2 Diabetes Mellitus Regardless of Lipid-Lowering Drug Use: A Cross-Sectional Cohort Study

**DOI:** 10.3390/nu15143270

**Published:** 2023-07-24

**Authors:** Tzu-Yuan Wang, Wei-Lun Chang, Cheng-Yu Wei, Chung-Hsiang Liu, Ray-Chang Tzeng, Pai-Yi Chiu

**Affiliations:** 1 Division of Endocrinology and Metabolism, Department of Internal Medicine, China Medical University Hospital, Taichung 404, Taiwan; yuan.w16@gmail.com; 2 Department of Neurology, Show Chwan Memorial Hospital, Changhua 500, Taiwan; a1977611@yahoo.com.tw; 3 Department of Exercise and Health Promotion, College of Kinesiology and Health, Chinese Culture University, Taipei 111, Taiwan; yuyu@seed.net.tw; 4 Department of Neurology, Chang Bing Show Chwan Memorial Hospital, Changhua 505, Taiwan; 5 Division of Department of Neurology, China Medical University Hospital, Taichung 404, Taiwan; greengen@gmail.com; 6 Department of Neurology, Tainan Municipal Hospital (Managed by Show Chwan Medical Care Corporation), Tainan 701, Taiwan; tzeng63@yahoo.com.tw; 7 Department of Applied Mathematics, Tunghai University, Taichung 407, Taiwan

**Keywords:** cholesterol paradox, type 2 diabetes mellitus, lipid-lowering drugs, cognition, “lower is better” strategy

## Abstract

Lipid-lowering drugs (LLDs) have protective effects against coronary artery disease (CAD) and cerebrovascular disease (CVD); however, a paradoxical association with cholesterol has been identified in several diseases, such as diabetes, dementia, and atrial fibrillation. We aimed to analyze the association between LLDs and cholesterol levels in older adults with type 2 diabetes mellitus (T2DM). This cross-sectional study enrolled consecutive patients aged ≥50 years from three centers in Taiwan. A multiple logistic regression model was used, and odds ratios (ORs) for different levels of total cholesterol (TC) or low-density-lipoprotein cholesterol (LDL-C) compared with the highest level were adjusted for age, triglyceride level, sex, comorbidities, and medications. Among the 3688 participants, 572 with and 676 without T2DM used LLDs. After adjusting for age and sex, the non-T2DM group demonstrated better medical conditions, cognition, and daily function than the T2DM group, regardless of LLD use. Compared to the highest TC level (≥240 mg/dL), ORs were significantly increased as TC levels decreased. A similar pattern of T2DM prevalence was observed in LDL-C levels. Older people with T2DM demonstrated low cognitive and daily functions. Significantly reduced TC and LDL levels were associated with a higher T2DM prevalence in older adults regardless of LLD use. T2DM was associated with impaired cognitive and daily functioning. A higher prevalence of T2DM in older people with low cholesterol levels raises doubt surrounding cognition and daily function being jeopardized when the “lower is better” strategy is applied for the secondary prevention of CAD or CVD.

## 1. Introduction

The “lower is better” strategy has become increasingly popular for clinically controlling low-density-lipoprotein cholesterol (LDL-C) levels in people at risk of coronary artery disease (CAD) or cerebrovascular disease (CVD). However, cholesterol is among the essential nutrients for maintaining good health, and many physiological functions depend on it, such as cell membrane structure [1], hormone production [2], myelin synthesis or repair [3], protection against viruses or endotoxins of bacteria [4], and many other functions that need cholesterol to maintain normal operations.

Over the past few decades, randomized controlled trials (RCTs) have provided robust evidence for the primary or secondary prevention of CAD or CVD by reducing LDL-C levels [5,6]. Additionally, a greater reduction in LDL-C may have a greater protective effect, even at very low or ultralow LDL-C levels [7,8]. Therefore, lowering the LDL-C levels has become the most acceptable strategy. However, controversial results are frequently mentioned in the subgroup analysis of these RCTs or real-world studies on the association of total cholesterol (TC), LDL-C levels, or lipid-lowering drugs (LLDs) with many other diseases, such as new-onset diabetes and cognitive impairment, as warned by the U.S. Food and Drug Administration, based on evidence indicating that LLD use or low cholesterol levels are not beneficial or may be harmful to these diseases [9].

The paradoxical association between cholesterol, including TC and LDL-C, and diabetes has been reported in several RCTs [10] and real-world studies [11]. A substantial percentage of new-onset diabetes, which is considered an adverse event, was observed in several RCTs [12,13]. For example, the Jupiter study has demonstrated a promising protective effect of rosuvastatin on CAD/CVD events; however, the risk of new-onset diabetes increased, and the hazard increased as LDL-C levels decreased [14]. A real-world study has demonstrated a U-shaped association between LDL-C levels and diabetes mellitus in patients with hypertension [15].

To elucidate these paradoxical findings, Nunes defined the “LDL cholesterol paradox” as a reduction in CAD/CVD risk related to a decrease in LDL-C blood levels which is not concomitant with a decrease in total mortality [16]. However, a paradoxical association between low cholesterol and new-onset atrial fibrillation (AF) has been reported in other studies. A Korean population study has reported that AF development was inversely associated with high lipid levels (for the top vs. bottom quartile; TC, hazard ratio (HR) 0.78, 95% confidence interval (CI) 0.76–0.81; LDL-C, HR 0.81, 95% CI 0.78–0.84; high-density lipoprotein cholesterol (HDL-C), HR 0.94, 95% CI 0.91–0.98; and TG, HR 0.88, 95% CI 0.85–0.92) [17]. An inverse association between cholesterol levels and AF has also been reported in many other studies [18,19,20,21].

The evidence of the paradoxical association between LLDs and new-onset diabetes in both RCTs and real-world studies is robust. However, whether older people are similar or more labile to the adverse effects of low LDL-C levels or LLDs remains unclear. Evidence has demonstrated old age as a crucial factor in new-onset diabetes. The PROSPER and WOSCOP trials are landmark studies on LLDs that have used pravastatin to prevent CAD/CVD events [22,23,24]. Participants in the WOSCOP group were significantly younger than those in the PROSPER group, and a discrepancy in new-onset diabetes was identified between the WOSCOP and PROSPER groups [25]. Another decisive factor associated with diabetes is low cholesterol level. A post hoc analysis of rosuvastatin in the Jupiter study has revealed that the risk of new-onset diabetes increased, and the risk increased as the LDL-C levels decreased [14].

Therefore, this study proposed that both age and low cholesterol levels are strongly associated with diabetes. We selected older people and investigated the association of TC or LDL-C levels with the prevalence of diabetes, hypothesizing that lower cholesterol levels, including TC and LDL-C, might be associated with diabetes in older people, and that LLDs might strengthen this effect if TC/LDL-C levels are ultralow.

## 2. Materials and Methods

### 2.1. Participants

**Figure 1:**
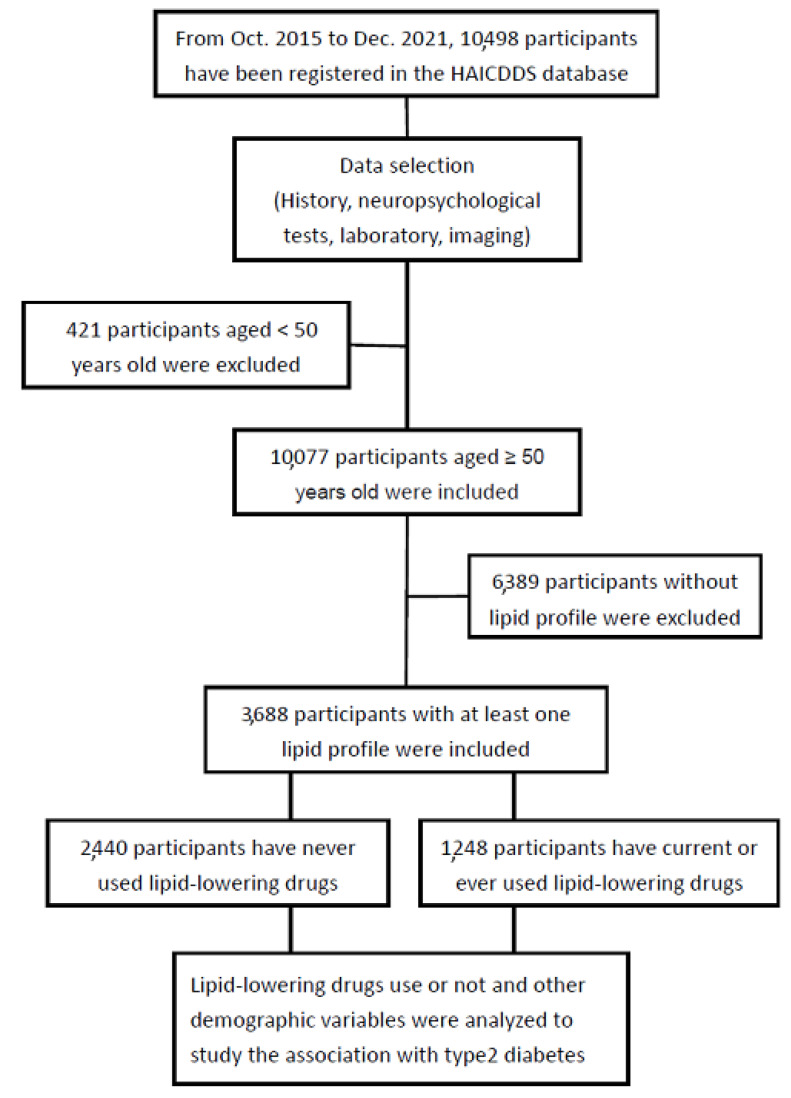
Flowchart of participant selection.

This was a cross-sectional cohort study, and a consecutive series of individuals aged at least 50 years from three centers in Taiwan were analyzed. LLD use was separately investigated to determine the association of TC or LDL-C levels with the prevalence of T2DM. T2DM was diagnosed if fasting blood glucose (FBG) was ≥126 mg/dL among undiagnosed participants or an International Classification of Diseases-10 code of T2DM with a claim for antidiabetic medication. We classified TC and LDL-C into different levels according to the usual recommendation for cholesterol control, particularly LDL-C based on the risks for further cardiovascular (CV) events according to several newer clinical criteria. Therefore, different TC/LDL-C levels were separately analyzed to study their association with diabetes. The flowchart of participant selection is presented in Figure 1.

### 2.2. Data Analysis

The Chinese version of SPSS (version 22.0; IBM Corp., Armonk, NY, USA) was used for the statistical analyses. The background characteristics of patients with diabetes were compared to those without diabetes according to use of lipid-lowering drugs, adjusting for age and sex. The LLD naïve and LLD use groups were compared in terms of cognition according to Montreal Cognitive Assessment (MoCA) [26], activities of daily living according to Instrumental Activities of Daily Living (IADL) scale [27], clinical history of hypertension, hyperlipidemia, CAD, arrhythmia, congestive heart failure (CHF), CVD, atherosclerosis, smoking, and exercise. The use of current medications, including antihypertensives, antidiabetes, antiplatelets, and anticoagulants, was compared. Medical measurements, including TC, LDL-C, HDL-C, triglycerides (TG), body mass index (BMI), systolic blood pressure (SBP), diastolic blood pressure, heart rate (HR), glycated hemoglobin (HbA1c), and FBG, were also compared.

A multiple logistic regression analysis was performed to investigate the contribution of TC and LDL-C levels to the T2DM prevalence. Odds ratios (ORs) for different levels compared to the highest (TC levels ≥ 240 or LDL-C levels ≥ 160) were adjusted for age, sex, MoCA, IADL, TG, CVD, hypertension, dyslipidemia, CAD, CHF, atherosclerosis, smoking, exercise, and use of antihypertensives, antiplatelets, and anticoagulants. All calculated *p*-values were two-tailed, and statistical significance was set at *p* < 0.05.

### 2.3. Ethical Consideration

This cohort study was conducted retrospectively, and the selected data were processed and analyzed anonymously. The Institutional Review Board of Show Chwan Memorial Hospital approved this study and waived the requirement for informed consent owing to the study design (SCMH_IRB No: IRB1110503).

## 3. Results

### 3.1. Background Information of the Patients

Among the 3688 participants, 572 of a total 1340 (42.7%) with T2DM and 676 of a total 2348 (28.8%) without T2DM used LLDs. Table 1 summarizes the background characteristics of the groups with and without diabetes, divided according to whether LLDs were used. After adjusting for age and sex, cognitive functions according to MoCA were better in those without diabetes (11.5 ± 8.2) than in those with diabetes (10.2 ± 7.6) among the non-LLD use group. A similar finding was observed in the LLD use group (13.2 ± 8.2 vs. 11.6 ± 7.5 in the non-T2DM group vs. the T2DM group) with both *p* < 0.001. Activities of daily living function according to IADL were better in those without diabetes (4.4 ± 3.3) than in those with diabetes (3.7 ± 3.2) among the non-LLD-use group, and a similar finding was observed in those with LLD use (5.0 ± 4.4 vs. 4.4 ± 3.2 in the non-T2DM group vs. the T2DM group) both with *p* < 0.001. In the drug-naïve group, the prevalence of almost all medical conditions, including hypertension, dyslipidemia, CAD, arrhythmia, CHF, CVD, and atherosclerosis, was higher in the T2DM group than in the non-T2DM group. The prevalence of medical conditions was significantly higher in the T2DM group than in the non-T2DM group with LLD use. The incidence of these medical conditions, including hypertension, dyslipidemia, and CVD, was lower in the T2DM group than in the non-T2DM group. Current medication use, including antihypertensives, antidiabetic drugs, antiplatelet agents, and anticoagulants, was higher in the T2DM group than in the non-T2DM group, regardless of LLD use. Lipid profiles, including TC, LDL-C, and HDL-C levels, were significantly lower in the T2DM group than in the non-T2DM group, regardless of LLD use. However, the TG, BMI, SBP, heart rate, fasting glucose, and HbA1c levels were significantly higher in the T2DM group than in the non-T2DM group, regardless of LLD use.

### 3.2. Percentage Frequencies of T2D According to the Different Levels of TC with or without LAAs

**Figure 2:**
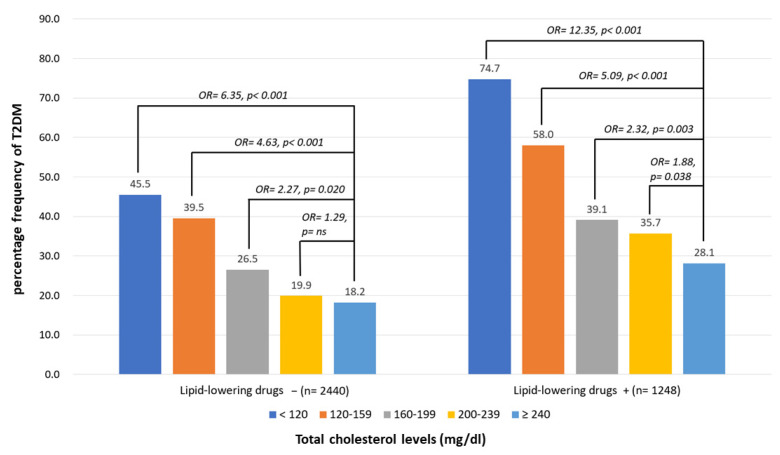
Percentage frequency of type 2 diabetes mellitus (T2DM) according to total cholesterol (TC) levels of participants with or without using lipid-lowering drugs. Multiple logistic regression analysis was adopted for investigating the contribution of TC levels to prevalence of T2DM. Odds ratios (ORs) for different levels compared to the highest (≥240) were adjusted for age, sex, Montreal Cognitive Assessment, Instrumental Activities of Daily Living, triglycerides, cerebrovascular diseases, hypertension, dyslipidemia, coronary artery diseases, congestive heart failure, atherosclerosis, smoking, exercise, and use of antihypertensives, antiplatelets, and anticoagulants. ns: nonsignificance, *p* > 0.05.

The percentage frequencies of diabetes in the drug-naïve group according to the different TC groups were 18.2%, 19.9%, 26.5%, 39.5%, and 45.5% in the >240, 200–239, 160–199, 120–159, and <120 mg/dL groups, respectively. A multiple logistic regression analysis was performed, and the ORs for different levels were adjusted for age, sex, MoCA, IADL, and related factors. Compared to the highest level of TC (>240 mg/dL), the ORs of the T2DM group according to different TC levels of the participants were 1.29 (*p* = ns), 2.27 (*p* = 0.020), 4.63 (*p* < 0.001), and 6.35 (*p* < 0.001) in the 200–239, 160–199, 120–159, and <120 mg/dL groups, respectively. The percentage frequencies of diabetes among the LLD-use group according to different TC levels were 28.1%, 35.7%, 39.1%, 58.0%, and 74.7% in the >240, 200–239, 160–199, 120–159, and <120 mg/dL groups, respectively. Compared to the highest TC level (>240 mg/dL) after adjustment, the OR of T2DM according to the different TC levels of the participants was 1.88 (*p* = 0.038), 2.32 (*p* = 0.003), 5.09 (*p* < 0.001), and 12.35 (*p* < 0.001) in the 200–239, 160–199, 120–159, and <120 mg/dL groups, respectively (Figure 2).

### 3.3. Percentage Frequencies of T2D According to the Different Levels of LDL-C with or without LAAs

**Figure 3:**
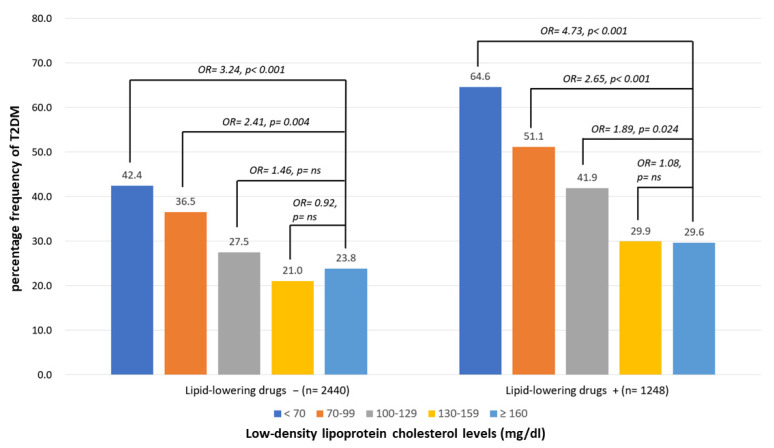
Percentage frequency of T2DM according to low-density-lipoprotein cholesterol (LDL-C) levels of participants with or without using lipid-lowering drugs. Multiple logistic regression analysis was adopted for investigating the contribution of LDL-C levels to prevalence of T2DM. Odds ratios (ORs) for different levels compared to the highest (≥160) were adjusted for age, sex, MoCA, IADL, triglyceride, cerebrovascular diseases, hypertension, dyslipidemia, coronary artery diseases, congestive heart failure, atherosclerosis, smoking, exercise, antihypertensives, lipid-lowering drugs, antiplatelets, and anticoagulants. *ns*: nonsignificance, *p* > 0.05.

The frequency of diabetes in the drug-naïve group according to different LDL-C levels was 23.8%, 21.0%, 27.5%, 36.5%, and 42.4% in the >160, 130–159, 100–129, 70–99, and <70 mg/dL groups, respectively. A multiple logistic regression analysis was performed, and the ORs for different levels were adjusted for age, sex, MoCA, IADL, and related factors. Compared to the highest level of LDL-C (>160 mg/dL), the ORs of T2DM according to different LDL-C levels of participants were 0.92 (*p* = ns), 1.46 (*p* = ns), 2.41 (*p* = 0.004), and 3.24 (*p* < 0.001), respectively. The percentage frequencies of diabetes among the LLD-use groups according to different TC levels were 29.6%, 35.7%, 41.4%, 51.1%, and 64.6% in the >160, 130–159, 100–129, 70–99, and <70 mg/dL groups, respectively. Compared to the highest level of LDL-C (>160 mg/dL) after adjustment, the ORs of the T2DM group according to different LDL-C levels of the participants were 1.08 (*p* = ns), 1.89 (*p* = 0.024), 2.65 (*p* < 0.001), and 4.73 (*p* < 0.001) in the 130–159, 100–129, 70–99, and <70 mg/dL groups, respectively (Figure 3).

### 3.4. Percentage Frequencies of T2D According to the Different Levels of TC in Different Centers

**Figure 4:**
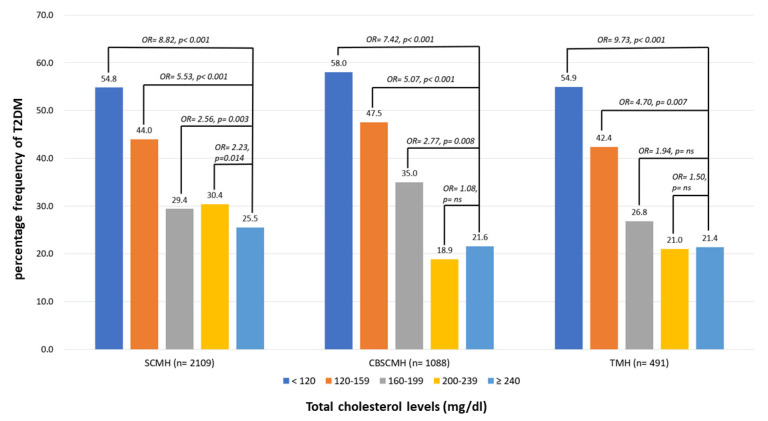
Percentage frequency of T2DM according to total cholesterol (TC) levels among participants in different centers. Multiple logistic regression analysis was adopted for investigating the contribution of TC levels to prevalence of T2DM. Odds ratios (ORs) for different levels compared to the highest (≥240) were adjusted for age, sex, MoCA, IADL, triglyceride, cerebrovascular diseases, hypertension, dyslipidemia, coronary artery diseases, congestive heart failure, atherosclerosis, smoking, exercise, antihypertensives, lipid-lowering drugs, antiplatelets, and anticoagulants. ns: nonsignificance, *p* > 0.05. SCMH: Show Chwan Memorial Hospital; CBSCMH: Chang Bin Show Chwan Memorial Hospital; TMH: Tainan Municipal Hospital.

Figure 4 demonstrates the percentage frequency of T2DM according to TC levels among participants in different centers, including Show Chwan Memorial Hospital (SCMH), Chang Bing Show Chwan Memorial Hospital (CBSCMH), and Tainan Municipal Hospital (TMH). The multiple logistic regression analysis with all relative factors adjusted demonstrated very similar association patterns of TC levels with diabetes, regardless of LLD use, among different centers.

### 3.5. Percentage Frequencies of T2D According to the Different Levels of LDL-C in Different Centers

**Figure 5:**
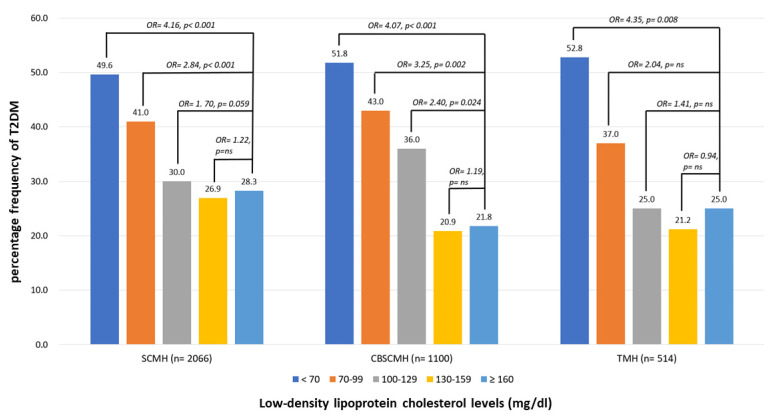
Percentage frequency of T2DM according to low-density lipoprotein cholesterol (LDL-C) levels among participants in different centers. Multiple logistic regression analysis was adopted for investigating the contribution of TC levels to prevalence of T2DM. Odds ratios (ORs) for different levels compared to the highest (≥160) were adjusted for age, sex, MoCA, IADL, triglyceride, cerebrovascular diseases, hypertension, dyslipidemia, coronary artery diseases, congestive heart failure, atherosclerosis, smoking, exercise, antihypertensives, lipid-lowering drugs, antiplatelets, and anticoagulants. ns: nonsignificance, *p* > 0.05. SCMH: Show Chwan Memorial Hospital; CBSCMH: Chang Bin Show Chwan Memorial Hospital; TMH: Tainan Municipal Hospital.

Figure 5 demonstrates the percentage frequency of T2DM according to LDL-C levels among participants in different centers, including SCMH, CBSCMH, and TMH. The multiple logistic regression analysis with all relative factors adjusted demonstrated very similar association patterns of LDL-C levels with diabetes, regardless of LLD use, among different centers.

## 4. Discussion

This study had several important findings. First, participants with diabetes had worse brain function, including cognitive and ADL functions. Individuals with diabetes tend to have more frequent associations with medical conditions, including vascular disorders, and medication use. Moreover, almost all the medical parameters of participants with diabetes were worse than those of participants without diabetes, except for TC and LDL-C levels, which were higher in the non-T2DM group. These findings were observed regardless of LLD use. Therefore, questions such as whether “lower LDL-C is better” is correct and why participants with diabetes have lower LDL-C levels instead of higher levels arise. Our findings on the paradoxical association between TC/LDL-C levels and diabetes in older people are consistent with those of RCTs or real-world studies that have suggested an increased risk of T2DM associated with the long-term use of LLDs in adults with or without CAD/CVD [10,11]. The pathophysiology of this phenomenon remains unclear; however, impaired β-cell function is a potential mechanism [28]. Other mechanisms involved in the association between LLDs and incident diabetes may include impaired Ca^2+^ signaling in pancreatic β-cells, the down-regulation of GLUT-4 in adipocytes, and compromised insulin signaling [29]. Additionally, the impact of statins on epigenetics potentially contributing to statin-induced T2DM via the differential expression of miRNAs has also been described [29]. Instead of LLD use, a stronger association with diabetes was observed in the drug-naive group. Several studies have suggested mechanisms, including alterations in insulin secretion, changes in ion channels, modulation of signaling pathways, and inflammation/oxidative stress, which are specific to beta cells [30]. PCSK9 deficiency reduces insulin secretion and promotes glucose intolerance. Moreover, PCSK9 loss-of-function genetic variants are associated with LDL-C, higher plasma glucose levels, and an increased risk of T2DM [31].

Furthermore, our original hypothesis of both old age and ultralow levels of cholesterol being crucial factors that influence the prevalence of T2DM were proved in our findings. Our findings on the influence of old age are consistent with those of RCTs that used the same drug in different age groups, such as the PROSPER and WOSCOP studies. The PROSPER study may be the first RCT to examine older adults with or without previous atherosclerotic CVD [22]. The PROSPER and WOSCOP trials [23] are both landmark LLD studies that used pravastatin to prevent CAD/CVD events [24]. The protocols of both trials were very similar, except that the participants in the WOSCOP trial were younger, with a mean age of 55 years, than those in PROSPER trial, with a mean age of 75 years. A discrepancy was identified in that new-onset diabetes in the WOSCOP trial was decreased, with an HR of 0.79 compared to placebo; however, that in the PROSPER trial was significantly increased, with an HR of 1.32 [25]. Moreover, our findings of the paradoxical association of TC or LDL-C levels with diabetes, regardless of LLDs, are also consistent with our proposed decisive factor of ultralow cholesterol levels. Evidence from a post hoc analysis in the Jupiter study has indicated that, compared with the placebo group, the risk of new-onset diabetes increased by 15% with LDL-C levels of >30 mg/dL using rosuvastatin and increased to 90% if the LDL-C was <30 mg/dL with the same drug [14].

This is a multicenter registration study that recruited the participants from three centers in Taiwan. The findings of a paradoxical association between TC and LDL-C levels were summarized among the centers, and the results are presented in Figure 5 separately. The results of the paradoxical association between cholesterol levels and diabetes were very similar in all three centers; therefore, we believe that the consistency is fair. Further large population studies with longitudinal follow-up to investigate not only the association between cholesterol level and new-onset diabetes but also their causal relationship are warranted.

The natural course of cholesterol level in humans increases after birth and decreases in the 50th and 60th decades of life [32]. Some clinicians or researchers may argue that, with LDL-C being the so-called bad cholesterol, its level should be as low as it is in the infant stage, especially in individuals with a high CVD risk. However, normal physiological functions, including cell membrane structure, myelination or neurotransmission in the nerves and brain, beta cell functions, hormone production, and many other functions, are all dependent on normal and sufficient cholesterol levels, either LDL or HDL; therefore, cholesterol levels continue to increase until the latter half of life [32]. Hence, the LDL-C level in the infant stage may not be sufficiently high to serve further physiological functions or deal with pathophysiological conditions. Finally, according to the findings of our study and those of several others, older people might be more susceptible to low TC or LDL-C levels and the adverse effects of LLDs. Robust evidence has demonstrated that the cholesterol paradox is ubiquitous, not only in diabetes or AF. Bad cholesterol may not always be bad, and further studies are warranted to investigate the balance between reducing and preserving LDL-C in controlling medical conditions such as CVD, CAD, dementia, diabetes, AF, infection, and many other diseases.

This study had some limitations. First, the comparison of the association between cholesterol levels and T2DM was cross-sectional; therefore, a causal relationship could not be established. Further longitudinal follow-up of a cohort that can trace new-onset diabetes among different cholesterol levels is warranted. Therefore, the contribution of TC, LDL-C, or LLDs may be possible. Second, our study was conducted in only two hospitals in Central Taiwan and one in Southern Taiwan. Therefore, selection bias was inevitable. Patients should be recruited from multiple centers to reduce selection bias in future studies. Third, although this study investigated the association between cognitive function and diabetes, the contribution of cholesterol and diabetes to cognitive decline could not be clarified. Further studies are warranted to investigate the causal relationships between diabetes, cholesterol levels, and cognitive impairment or dementia.

## 5. Conclusions

In conclusion, significantly reduced TC or LDL-C levels were associated with a higher prevalence of T2DM in older adults, regardless of LLD use. The higher prevalence of T2DM in those who never used LLDs with low cholesterol levels makes the “lower is better” strategy for the secondary prevention of CAD or CVD controversial. Moreover, older people with T2DM demonstrated lower cognitive and daily functions. A higher prevalence of T2DM in older people with low cholesterol levels will also raise doubt surrounding cognition and daily function being jeopardized when the “lower is better” strategy is applied for preventing cognitive impairment or dementia.

## Figures and Tables

**Table 1:** Background characteristics of the patients with diabetes compared to those without diabetes divided according to use of lipid-lowering drugs, adjusted for age and sex.

	Lipid-Lowering Drug −			Lipid-Lowering Drug +, Mean (SD)		
**Group**	Diabetic	Nondiabetic	OR, *p*	Diabetic	Nondiabetic	OR, *p*
***n***	768	1672		572	676	
**Age, year, mean (SD)**	76.2 (8.7)	76.1 (9.6)	-	74.2 (8.9)	74.1 (9.6)	-
**Male, *n* (%)**	336 (43.8)	755 (45.2)	-	232 (40.6)	293 (43.3)	-
**MoCA, mean (SD)**	10.2 (7.6)	11.5 (8.2)	0.97, <0.001	11.6 (7.5)	13.3 (8.2)	0.97, <0.001
**IADL, mean (SD)**	3.7 (3.2)	4.4 (3.3)	0.95, <0.001	4.4 (3.2)	5.0 (4.4)	0.93, <0.001
**Clinical history, *n* (%)**						
**Hypertension**	609 (79.3)	966 (57.8)	2.81, <0.001	493 (86.2)	527 (78.0)	1.77, <0.001
**Dyslipidemia**	304 (39.6)	295 (17.6)	3.10, <0.001	438 (76.6)	466 (68.9)	1.47, 0.003
**CAD**	91 (11.8)	138 (8.3)	1.50, <0.001	80 (14.0)	88 (13.0)	1.10, ns
**Arrythmia**	127 (16.5)	223 (13.3)	1.28, 0.039	120 (21.0)	119 (17.6)	1.25, ns
**CHF**	66 (8.6)	97 (5.8)	1.52, 0.011	57 (10.0)	64 (9.5)	1.06, ns
**CVD**	333 (43.4)	530 (31.7)	1.67, <0.001	249 (43.5)	261 (38.6)	1.26, 0.048
**Atherosclerosis**	220 (28.6)	408 (24.4)	1.25, 0.023	238 (41.6)	337 (49.9)	0.72, 0.005
**Smoking**	165 (21.5)	374 (22.4)	0.98, ns	122 (21.3)	133 (19.7)	1.29, ns
**Exercise**	177 (23.0)	502 (30.0)	0.69, <0.001	165 (27.1)	228 (33.7)	0.73, 0.012
**Current medication, *n* (%)**						
**Antihypertensives**	325 (42.3)	625 (37.4)	1.23, 0.021	461 (80.6)	480 (71.0)	1.70, <0.001
**Antidiabetes**	331 (43.1)	4 (0.2)	320.24, <0.001	492 (86.0)	18 (2.7)	235.78, <0.001
**Antiplatelets**	326 (42.4)	573 (34.3)	1.43, <0.001	412 (72.0)	438 (64.8)	1.42, 0.005
**Anticoagulants**	71 (9.2)	95 (5.7)	1.69, 0.001	96 (16.8)	86 (12.7)	1.40, 0.038
**Medical measurement** **, mean (SD)**						
**TC, mg/dL**	158.3 (33.4)	171.8 (35.5)	0.99, <0.001	184.6 (40.6)	164.8 (40.4)	0.99, <0.001
**LDL-C, mg/dL**	94.5 (29.3)	104.1 (30.5)	0.99, <0.001	112.2 (36.7)	96.6 (34.4)	0.99, <0.001
**HDL-C, mg/dL**	47.9 (14.9)	53.0 (15.5)	0.98, <0.001	48.8 (14.9)	54.1 (15.7)	0.97, <0.001
**TG, mg/dL**	129.5 (74.8)	113.2 (65.6)	1.03, <0.001	150.4 (96.6)	132.8 (90.3)	1.02, <0.001
**BMI, kg/m^2^**	24.9 (4.0)	23.5 (3.4)	1.12, <0.001	25.2 (4.1)	24.4 (3.9)	1.06, <0.001
**SBP, mmHg**	134.8 (20.2)	133.5 (18.5)	1.00, ns	135.9 (17.9)	135.1 (18.2)	1.00, ns
**DBP, mmHg**	71.8 (12.8)	73.0 (12.3)	0.99, ns	73.3 (12.7)	74.9 (12.4)	0.99, 0.042
**HR, bpm**	80.8 (13.8)	78.2 (13.0)	1.02, 0.002	80.4 (13.5)	78.1 (13.2)	1.02, 0.002
**Fasting glucose, mg/dL**	152.2 (88.9)	103.0 (24.1)	1.03, <0.001	144.1 (61.4)	103.1 (22.2)	1.04, <0.001
**HbA1C, mg/dL**	6.9 (1.4)	5.6 (0.5)	7.78, <0.001	7.0 (1.5)	5.8 (0.8)	5.97, <0.001

*n*: number of cases; ns: nonsignificance; OR: odds ratio; MoCA: Montreal Cognitive Assessment; IADL: Instrumental Activities of Daily Living scale; CAD: coronary artery disease; CHF: congestive heart failure; CVD: cerebrovascular disease; TC: total cholesterol; LDL-C: low-density-lipoprotein cholesterol; HDL-C: high-density-lipoprotein cholesterol; TG: triglycerides; BMI: body mass index; SBP: systolic blood pressure; DBP: diastolic blood pressure; HR: heart rate; HbA1c: Glycated Hemoglobin.

## Data Availability

All data and material are included in the manuscript.
